# Insights into the origin of rare haplogroup C3* Y chromosomes in South America from high-density autosomal SNP genotyping

**DOI:** 10.1016/j.fsigen.2014.11.005

**Published:** 2015-03

**Authors:** Massimo Mezzavilla, Maria Geppert, Chris Tyler-Smith, Lutz Roewer, Yali Xue

**Affiliations:** a The Wellcome Trust Sanger Institute, Wellcome Trust Genome Campus, Hinxton, Cambridgeshire CB10 1SA, UK; b Medical Genetics, Department of Reproductive Sciences and Development, IRCCS-Burlo Garofolo, University of Trieste, Trieste, Italy; c Department of Forensic Genetics, Institute of Legal Medicine and Forensic Sciences, Charité-Universitätsmedizin, Berlin, Germany

**Keywords:** Past human migrations, Ecuador, Admixture, Simulations

## Abstract

The colonization of Americas is thought to have occurred 15–20 thousand years ago (Kya), with little or no subsequent migration into South America until the European expansions beginning 0.5 Kya. Recently, however, haplogroup C3* Y chromosomes were discovered in two nearby Native American populations from Ecuador. Since this haplogroup is otherwise nearly absent from the Americas but is common in East Asia, and an archaeological link between Ecuador and Japan is known from 6 Kya, an additional migration 6 Kya was suggested. Here, we have generated high-density autosomal SNP genotypes from the Ecuadorian populations and compared them with genotypes from East Asia and elsewhere to evaluate three hypotheses: a recent migration from Japan, a single pulse of migration from Japan 6 Kya, and no migration after the First Americans. First, using forward-time simulations and an appropriate demographic model, we investigated our power to detect both ancient and recent gene flow at different levels. Second, we analyzed 207,321 single nucleotide polymorphisms from 16 Ecuadorian individuals, comparing them with populations from the HGDP panel using descriptive and formal tests for admixture. Our simulations revealed good power to detect recent admixture, and that ≥5% admixture 6 Kya ago could be detected. However, in the experimental data we saw no evidence of gene flow from Japan to Ecuador. In summary, we can exclude recent migration and probably admixture 6 Kya as the source of the C3* Y chromosomes in Ecuador, and thus suggest that they represent a rare founding lineage lost by drift elsewhere.

## Highlights

• Revisited the previous discovery of a rare Y haplogroup in two Ecuador populations.
• Hypotheses for the origin of the haplogroup tested with autosomal SNP genotype data.
• We favoured one of the three hypotheses, ‘founder plus drift’.

## 1 Introduction

The consensus view of the peopling of the Americas, incorporating archaeological, linguistic and genetic evidence, proposes colonization by a small founder population from Northeast Asia via Beringia 15–20 Kya (thousand years ago), followed by one or two additional migrations also via Alaska, contributing only to the gene pools of North Americans, and little subsequent migration into the Americas south of the Arctic Circle before the voyages from Europe initiated by Columbus in 1492 [1]. In the most detailed genetic analysis thus far, for example, Reich and colleagues [2] identified three sources of Native American ancestry: a ‘First American’ stream contributing to all Native populations, a second stream contributing only to Eskimo-Aleut-speaking Arctic populations, and a third stream contributing only to a Na-Dene-speaking North American population. Nevertheless, there is strong evidence for additional long-distance contacts between the Americas and other continents between these initial migrations and 1492. Norse explorers reached North America around 1000 CE and established a short-lived colony, documented in the Vinland Sagas and supported by archaeological excavations [3]. The sweet potato (*Ipomoea batatas*) was domesticated in South America (probably Peru), but combined genetic and historical analyses demonstrate that it was transported from South America to Polynesia before 1000–1100 CE [4]. Some inhabitants of Easter Island (Rapa Nui) carry HLA alleles characteristic of South America, most readily explained by gene flow after the colonization of the island around 1200 CE but before European contact in 1722 [5]. In Brazil, two nineteenth-century Botocudo skulls carrying the mtDNA Polynesian motif have been reported, and a Pre-Columbian date for entry of this motif into the Americas discussed, although a more recent date was considered more likely [6]. Thus South America was in two-way contact with other continental regions in prehistoric times, but there is currently no unequivocal evidence for outside gene flow into South America between the initial colonization by the ‘First American’ stream and European contact.

The Y chromosome has a number of advantages for studies of human migrations. Haplotypes over most of its length are male-specific and evolve along stable lineages only by the accumulation of mutations, and the small male effective population size results in high levels of genetic drift of these haplotypes [7]. Consequently, major Y lineages (haplogroups) are often specific to particular geographical areas, and migrant haplogroups may stand out because they are atypical of the place where they are found (e.g. [8,9]). The most comprehensive study of indigenous South American Y chromosomes thus far surveyed 1011 individuals and found that while most of them belonged to haplogroup Q as expected, 14 individuals from two nearby populations in Ecuador carried haplogroup C3*(xC3a-f) chromosomes (henceforth C3*), with this haplogroup reaching 26% frequency in the Kichwa sample and 7.5% in the Waorani [10]. The estimated TMRCA for the combined Ecuadorian C3* chromosomes was 5.0–6.2 Kya. The finding of this haplogroup in Ecuador was surprising because C3* is otherwise unreported from the Americas (apart from one example in Alaska), but is widespread and common in East Asia.

**Fig. 1:**
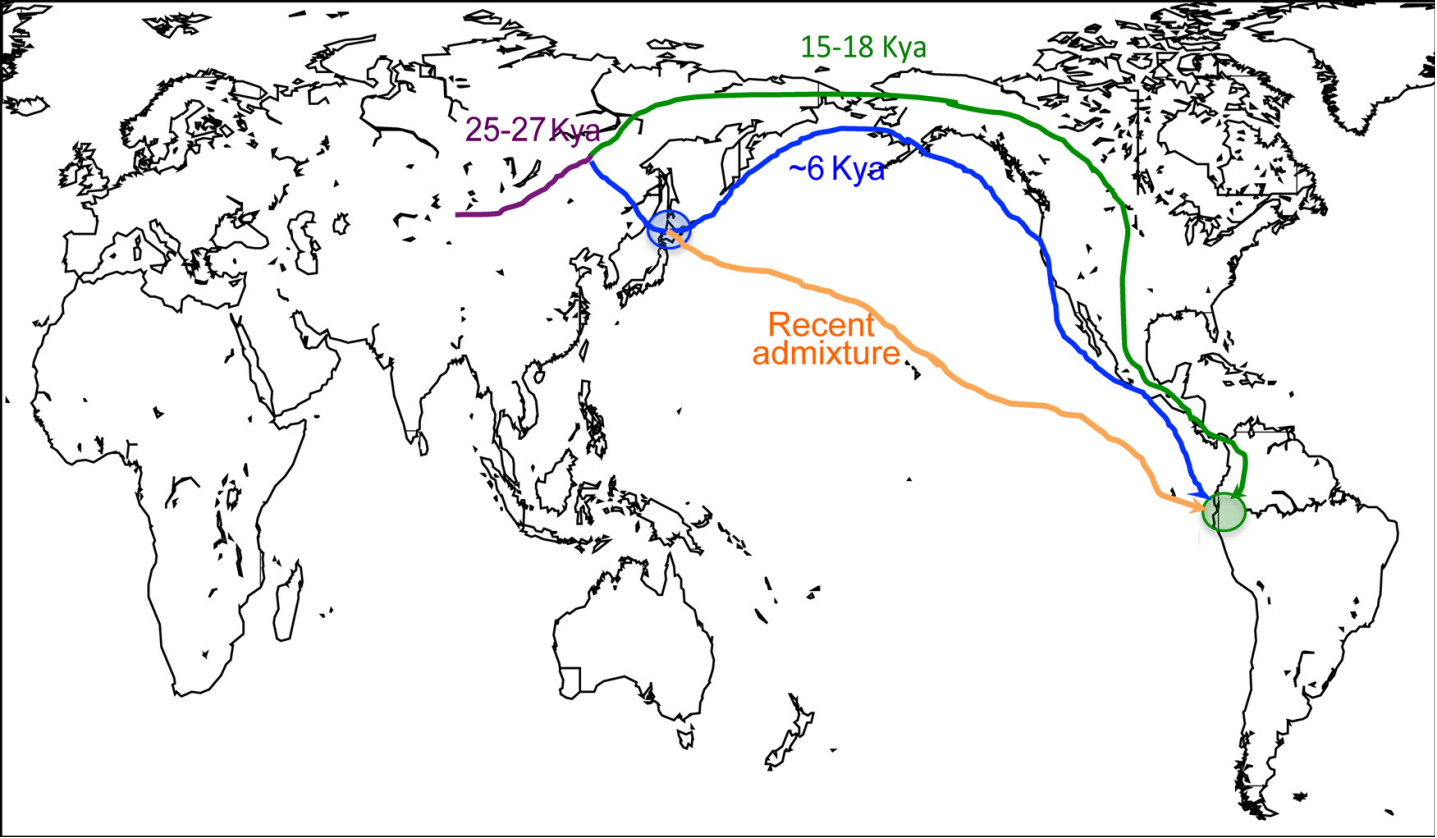
Hypotheses to explain the presence of C3* Y chromosomes in Ecuador but not elsewhere in the Americas. Green line: the chromosomes were carried by the First American founder population 15–20 Kya, but have been lost by drift from other present-day Native American populations examined. Blue line: they were introduced from Japan by a migration identified by archaeologists around 6 Kya. Orange line: they were introduced from East Asia in the last generation or two. The violet line represents an ancestral population that split around 25–27 Kya into the ancestors of present-day East Asians and Native Americans, and is incorporated into the demographic model used in this work.

Three scenarios might explain the presence of C3* chromosomes at a mean frequency of 17% in these two Ecuadorian populations [10], Fig. 1. First, they might represent recent admixture with East Asians during the last few generations. This possibility was considered unlikely because the Waorani discouraged contact with outsiders using extreme ferocity until peaceful links were established in 1958, and known male ancestors (fathers, grandfathers) of C3* carriers were born before this date. Second, C3* might have been another founding lineage entering the Americas 15–20 Kya, and have drifted down to undetected levels in all populations examined except the Ecuadorians. This was also considered unlikely because the populations of North and Central America have in general experience less drift and retained more diversity than those in South America [2], and so it would be surprising to lose C3* from North/Central Americans but not South Americans. Third, C3* could have been introduced into Ecuador from East Asia at some intermediate date by a direct route that bypassed North America. In support of this third scenario, archaeologists have identified similarities in pottery between the middle Jōmon culture of Kyushu (Japan) and the Valdivia culture of coastal Ecuador dating to 5.3–6.4 Kya; notably, like the C3* chromosomes, such a ceramic complex in the Americas was unique to Ecuador and was not reported from North or Central America or elsewhere from South America [11]. We refer to these three scenarios as ‘recent admixture’, ‘founder plus drift’ and ‘ancient admixture’, respectively.

In this follow-up study, we set out to revisit the three hypotheses for the origin of the C3* Y chromosomes in Ecuador. One possibility would be to sequence the Ecuadorian C3* Y chromosomes, and compare them with existing or additional East Asian C3* chromosome sequences, to determine the divergence time. However, the limited quantity and quality of DNA available did not allow this. We therefore followed another possibility, using genome-wide autosomal SNP genotyping. By comparing the autosomal genotypes of the Kichwa and Waorani samples with other populations including Native South Americans and East Asians, we expect that admixture in the last few generations (‘recent admixture’) would be readily detectable, and admixture ∼6 Kya potentially detectable (‘ancient admixture’), from the presence of East Asian autosomal segments only found in the Ecuadorian samples compared with the other Native South Americans. This would allow discrimination between these hypotheses. With whole genome autosomal data, we could investigate the whole population samples of both females and males, including either carriers of C3* chromosomes or of other Y haplogroups, since any admixture would affect the whole population.

## 2 Materials and methods

### 2.1 DNA samples

31 samples from Ecuador (12 male and 10 female Waorani, 7 male and 2 female Kichwa) with DNA concentrations between 0.05 and 1 ng/μl were chosen for whole-genome amplification (WGA). Between 10 and 25 μl (depending on the DNA concentration) were concentrated in a Speed Vac (Thermo Scientific) to increase the DNA concentration to at least 1 ng/μl. Afterwards the samples were whole-genome amplified using the Illustra GenomiPhi HY DNA Amplification Kit (GE Healthcare). The protocol was adapted to a final volume of 20 μl as follows: 1 μl of the sample DNA and 9 μl sample buffer were mixed and denatured for 3 min at 95 °C. Samples were then cooled to 4 °C. In the next step, 9 μl reaction buffer and 1 μl enzyme mix were added to the sample and incubated as 30 °C for 4 h. The WGA reaction was inactivated at 65 °C for 10 min. 11 JPT samples with concentration of 10 ng/μl [12] were amplified using the same protocol.

The quality of the resulting DNA was tested by a PCR reaction using the AmpFlSTR^®^ NGM™ PCR Amplification Kit. WGA samples were diluted 100 or 200 times depending on initial DNA concentration. For all WGA-treated samples, full profiles were obtained which were concordant with the DNA profiles of the original unamplified sample DNA.

This study, together with the informed consent, was approved by the ethics committee of the Institute of Legal Medicine and Forensic Sciences (Charité-Universitätsmedizin, Berlin, Germany) under the accession number 11-2010/02.

### 2.2 Genotyping and quality control

31 individuals from Ecuador (22 Waorani and 9 Kichwa) and 11 from Japan (JPT) were genotyped using the Illumina HumanOmni2.5-8 (Omni2.5) BeadChip. Genotypes across these samples were called using Gencall (http://www.illumina.com/Documents/products/technotes/technote_gencall_data_analysis_software.pdf) via the Sanger standard genotype-calling pipeline, then merged with available genotypes from the HGDP population panel [13]. We then removed individuals with a low genotyping rate (>20% missing data) and with high relatedness (PI_HAT > 0.5); for the HGDP data, we used the subset of individuals recommended [14]. The final dataset consists of 207,321 single nucleotide markers (SNPs) with an average genotyping rate >99.7% in 967 individuals. After QC there were 16 individuals from Ecuador (*Supplementary* Table 1) and 11 JPT; the HGDP panel contained an additional 39 Japanese individuals. All analyses were performed using PLINK v.107 [15]. The number of individuals from each population is reported in *Supplementary* Table 2.

### 2.3 Simulations

Simulations were used to assess the power to detect ancient or recent admixture. In all our simulations we used unlinked markers for two reasons: first, the main analyses used were ADMIXTURE [16], the three-population test [17], TREEMIX [18] and Principal Components Analysis (PCA), which all assume unlinked markers; second, the probability to find a segment of *x* cM (from the source population) *λ* generations after admixture is 1 − (1 − *e*^(−*λx*)^), so we estimated that 90% of the fragments remaining after 6.000 years would be shorter than 50 kb, so considering the level of linkage disequilibrium could be considered as single loci.

One simulation approach was used to estimate the minimum threshold of recent admixture that would be detectable. We selected 5000 unlinked markers from the JPT and Ecuadorian SNP genotypes, and created artificial genomes with different levels of markers coming from one population. In detail, we simulated 16 admixed Ecuadorians with 50%, 20%, 10%, 5% or 1% JPT admixture; the simulated admixed individuals were then analyzed using ADMIXTURE v.122 with Ecuadorian and Japanese as reference populations.

Simulations to evaluate the power to detect a single more ancient admixture event were performed using the simuPOP python library [19], using parameter values for effective population size and populations split times obtained from the SNP genotype data using the procedure of McEvoy [20] implemented in the NeON R package available at http://www.unife.it/dipartimento/biologia-evoluzione/ricerca/evoluzione-e-genetica/software. We modelled a single pulse of migration from a Source population (representing the East Asian population) to produce an Admixed population (representing the Ecuadorian population); an additional population was simulated as a control (representing an unmixed Native American population). The probability for one individual to migrate from the Source population to the Admixed population was set at 0%, 1%, 5% or 10%. For the 10% scenario, individuals were sampled before the migration event, immediately after the migration event, and at the present time, 6 Ky later. The sample size used was 50 individuals, the genome considered consisted of 2200 independent loci on 22 chromosomes; each scenario was replicated 100 times. Each replicated dataset was analyzed using ADMIXTURE v.122.

### 2.4 Population structure

Principal Components Analysis was carried out using EIGENSOFT v.5.0.2 [21]. Ecuadorian and JPT samples were projected onto the axes obtained from all HGDP populations. PCA was performed on two different datasets: first, with all the populations in this study, and second with just the Native Americans (including the Ecuador samples), Japanese (including JPT), Yakut, French and Russian samples.

### 2.5 Admixture and migration

Analysis of admixture components was performed using ADMIXTURE v.1.22 applied to the two different datasets described in Section 2.4 above. Three different approaches were used to search for evidence of migration into the Ecuadorian population: first, the three-population test [17], second, the maximum-likelihood tree approach implemented in TREEMIX v.1.1 [18] (performed on the two datasets) considering from 0 to 12 migration events; and third, a method based on the decay of linkage disequilibrium implemented in ALDER v 1.03, which also provides an estimate of the time of admixture [22].

## 3 Results

We first used simulations to evaluate our power to detect recent admixture (in the last few generations) or more ancient admixture (∼6 Kya) as suggested in the previous study [10], compared with a non-admixed population established 15–20 Kya. Then we examined newly-generated data from the Ecuadorian population to determine whether or not any admixture was detectable.

### 3.1 Power to detect admixture

**Fig. 2:**
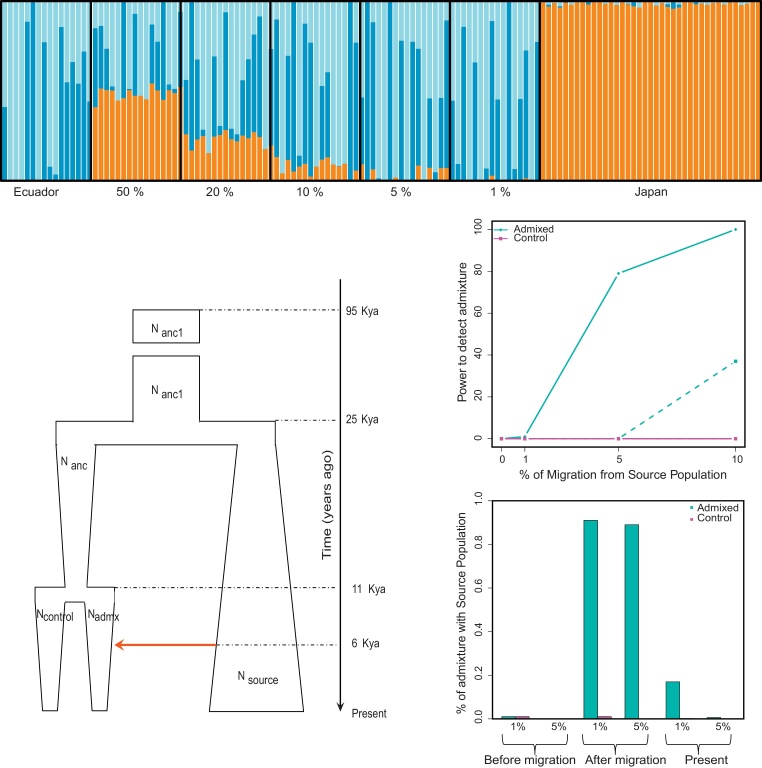
Evaluation by simulation of the power to detect recent or 6 Kya Japanese admixture in the Ecuadorian population. (A) Simulation of recent admixture: ADMIXTURE analysis of artificially admixed Ecuadorian and Japanese genomes. For the optimal value of *K* (*K* = 3), the Japanese component (orange) is absent from the Ecuadorian population, but is present in some or all individuals from the admixed populations with admixture levels of 50%, 20%, 10%, 5% or 1%. (B)–(D) Simulation of admixture 6 Kya. (B) Demographic model used in the simulations. We considered one population of constant size for 70 Ky from 95 Kya to 25 Kya (with size *N*_anc1_). At 25 Kya, this split into two. One daughter population (*N*_source_) expanded and represents the Source population. The other split into two 11 Kya, representing the Control population (*N*_control_) and the Admixed population (*N*_admix_). A single pulse of migration occurred 6 Kya (red arrow) from the Source to the Admixed population, with the level of admixture varied in different simulations. (C) Power to detect admixture that occurred 6 Kya in present-day samples. Migration 6 Kya was set at 0%, 1%, 5% or 10%. The cyan continuous line shows the power to detect a mean level ≥0.1% admixture in the present-day Admixed population, and the cyan dotted line the power to detect ≥0.5% admixture. The magenta lines show the corresponding detection in the Control population. (D) The fraction of simulated individuals from the scenario with 10% migration 6 Kya who have an admixture level greater than 1% or 5%, sampled before the migration, one generation after, or 6 Ky after in the present-day population: cyan, in the Admixed population; magenta, in the control population.

For the recent admixture model, we found that we could detect ∼50% or ∼20% of Japanese ancestry in all the individuals in the 50% or 20% artificial admixed simulations, respectively. With lower proportions of admixture, there was more variation between individuals, but we identified 3–14% Japanese ancestry in all but one individual in the 10% artificial admixed simulation. We detected 1–9% of Japanese ancestry in about half of the individuals in the 5% artificial admixed simulations, and 1–2% in two individuals in the simulations of 1% artificial admixture (Fig. 2A). So we are well-powered for detecting recent admixture, and detect it in some individuals from a population sample of 16 even at 1% admixture.

We then simulated a scenario where the admixture had occurred 6 Kya, using the demographic parameters estimated from the linkage disequilibrium pattern as described previously [20], shown in Fig. 2B and *Supplementary* Table 2. A single pulse of migration was set at 0%, 1%, 5% and 10%. Due to genetic drift in the relatively small population, after 6 Ky the population average level of admixture in the present-day population was much less than the starting amount. The power to detect ancient admixture at these levels therefore depends on the sensitivity to detect the reduced admixture in the present-day population. For example, if 0.1% mean population admixture can be detected in the present-day population, we have ∼80% power to detect 5% ancient admixture and ∼100% power to detect 10% ancient admixture. If, instead, we could only detect 0.5% mean admixture in the present-day population, we have ∼0 power to detect 5% ancient admixture and ∼35% power to detect 10% ancient admixture (Fig. 2C). With these population mean levels of admixture, the admixture in different individuals in the population can vary substantially. Immediately after a pulse of 10% migration, almost all individuals in the Admixed population have >5% and >1% admixture (Fig. 2D, middle section), as also seen in Fig. 2A. 6 Ky later, in the present-day population, about 1% of the Admixed individuals retain 5% admixture, and about 17% retain 1% admixture (Fig. 2d, right-hand section). In all simulations, negligible admixture is detected in the Control population. Overall, we have good power to detect 10% admixture that took place 6 Kya, and some power to detect 5% ancient admixture.

### 3.2 Population structure, admixture and migration patterns observed in Ecuadorians

**Supplementary Fig. 2:**
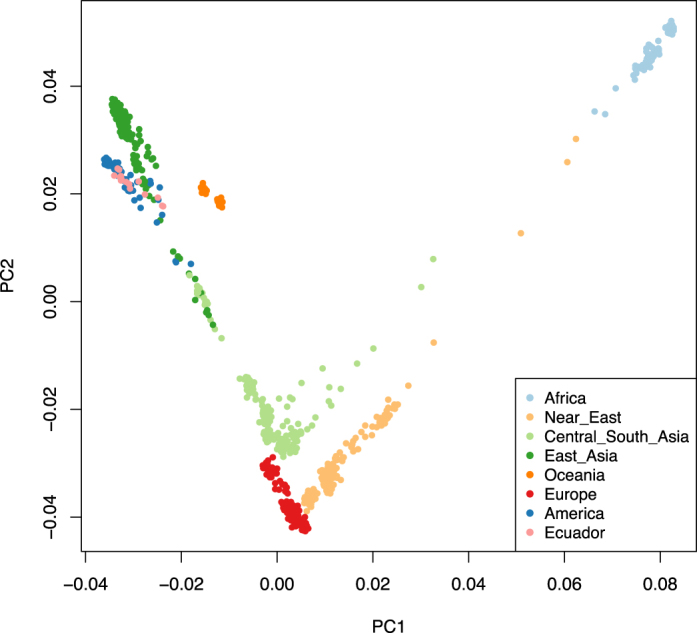
Principal Components Analysis of the HGDP dataset plus the new JPT and Ecuadorian samples. Principal Components Analysis was carried with EIGENSOFT, and Ecuadorian and JPT samples were projected on the axes obtained using all HGDP populations.

**Supplementary Fig. 3:**
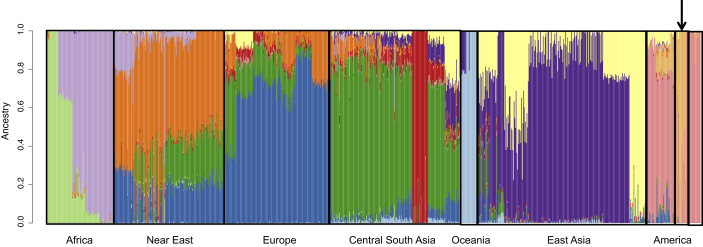
ADMIXTURE analysis with all HGDP populations. The lowest cross-validation error was obtained for *K* = 11, shown here. The black arrow indicates the Ecuadorian population, which at *K* = 11 shows its predominant unique cluster (light orange).

**Fig. 3:**
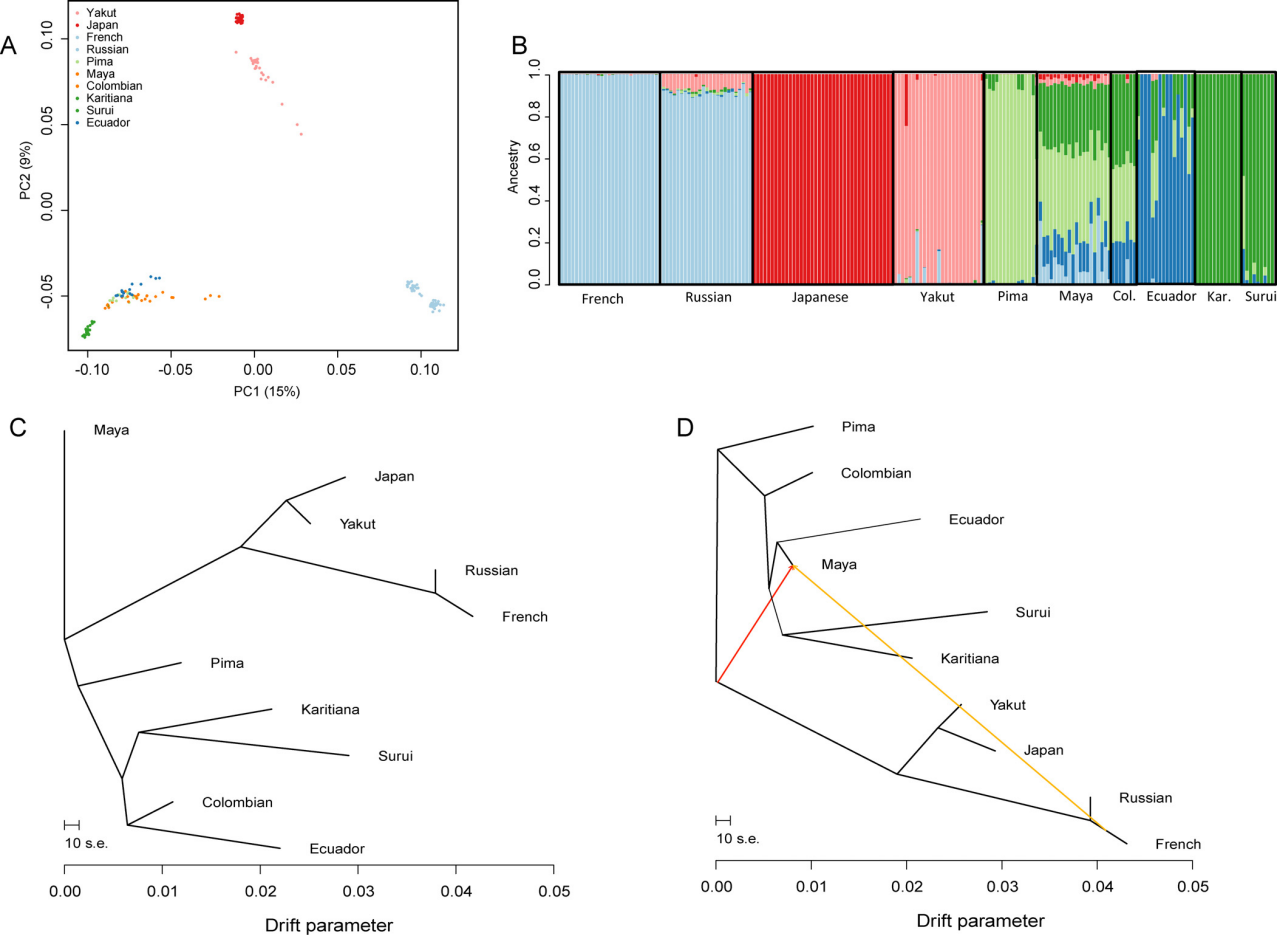
Analysis of admixture and migration in the Ecuadorian, Japanese, and other relevant populations. (A) Principal Components Analysis of a focused dataset consisting of the Ecuadorians, two European populations, two East Asian populations, and all the Native American populations in the HGDP panel. (B) Admixture plot for the same populations with *K* = 6. The Japanese cluster was not detected in the Ecuadorians. (C, D) Migration graphs generated using TREEMIX. (C) TREEMIX with no migration. (D) TREEMIX with the maximum supported number of migration events (two) reveals two migration events into the Maya, but no evidence of migration into the Ecuadorians.

We genotyped the available Ecuadorian samples at ∼2.5 M sites. The quality of the DNA was low, so it was necessary to perform whole-genome amplification, and even after this step only about half (16/31) of the samples passed QC. For comparison, we analyzed 11 whole-genome amplified JPT samples in parallel. In order to assess the results, we first compared the genotypes with those of the HGDP populations, using either the worldwide set, or a subset focussed on the relevant populations. Worldwide (*Supplementary* Fig. 2) and focused PCA (Fig. 3A) both showed that the amplified JPT fell among the HGDP Japanese, indicating that the amplification procedure and different genotyping chip and centre had no effect detectable by this analysis. Similarly, the Ecuadorians grouped with other Native American populations. ADMIXTURE analyses supported these findings, although with the Ecuadorians tending to form their own cluster at the optimal value of K (*Supplementary* Fig. 3; Fig. 3B). Some Ecuadorian individuals showed evidence of ancestral components shared with other Native American populations, visible, for example, as the mid green and light green components in Fig. 3B. In addition, in the focussed analysis, two Ecuadorians showed around 5% of a pink ancestral component most prevalent in the Yakut. This component was also detectable at a low level in some Colombians and all of the Maya, as well as in the Russians, so may represent widespread ancient shared ancestry. None of the Ecuadorians showed any of the red component characteristic of the Japanese. This red component was, however, detectable in most of the Maya.

While PCA and ADMIXTURE provide a useful visualization of the data, we also performed more formal tests for admixture. TREEMIX again grouped the Ecuadorians with other Native American populations (Fig. 3C), and when migration was included in the model, the only migration events supported in the focussed group of populations were two events in the Maya (Fig. 3D). We then ran the three-population test and ALDER analysis with all possible population combinations, using Ecuador as a target. No significant results were obtained for either of these two analyses (Table 1), showing that there is no support for migration into the Ecuadorian population.

## 4 Discussion

We set out to test whether or not the haplogroup C3* Y chromosomes found at a mean frequency of 17% in two Ecuadorian populations [10] could have been introduced by migration from East Asia, where this haplogroup is common. We considered recent admixture in the last few generations and, based on an archaeological link between the middle Jōmon culture in Japan and the Valdivia culture in Ecuador [11], a specific example of ancient admixture between Japan and Ecuador 6 Kya (Fig. 1).

Simulations of recent admixture, and ancient admixture based on a demographic model of the relevant populations (Fig. 2B), revealed that we had good power to detect 1% recent admixture and 10% ancient admixture, with some power to detect 5% ancient admixture (Fig. 2). The lower power to detect ancient admixture was due to the extensive drift in the small Native American populations providing opportunities for the admixture signal to be lost by chance. No evidence for admixture was found in the autosomal SNP genotype data (Fig. 3, Table 1).

Since the C3* Y chromosomes are present in the Ecuadorian populations at moderate frequency, the absence of evidence for >1% recent admixture is strong evidence against their recent introduction into Ecuador. It is more difficult to rule out ancient admixture. While no such admixture was detected, it remains possible that ancient admixture occurred at a low level (e.g. 1%), the introduced Y chromosomes then drifted up in frequency to their present level, and the introduced autosomal segments remained at, or drifted down to, undetectable levels. Nevertheless, the simplest interpretation of our results is that there was no ancient admixture, and the explanation for the presence of the C3* Y chromosomes in Ecuador must lie elsewhere. The remaining scenario is the ‘founder plus drift’ model (Fig. 1). With this model, the difficulty is to explain why the generally more genetically diverse North and Central American populations lack C3* Y chromosomes, while the less diverse South American populations retain them. Future simulations can be used to address this issue, and C3* Y chromosome with potential North/Central Native American affiliations should be evaluated carefully. Ancient DNA samples would be particularly relevant. In addition, as indicated in the Introduction, an attractive approach would be to sequence modern Ecuadorian and Asian C3* Y chromosomes and estimate the divergence time [23]: a time >15 Kya would support the founder plus drift model, while a time of 6 Kya or slightly higher would support the specific ancient admixture model considered here. Additional Ecuadorian DNA samples will be required for this.

## 5 Conclusions

Three different hypotheses to explain the presence of C3* Y chromosomes in Ecuador but not elsewhere in the Americas were tested: recent admixture, ancient admixture ∼6 Kya, or entry as a founder haplogroup 15–20 Kya with subsequent loss by drift elsewhere. We can convincingly exclude the recent admixture model, and find no support for the ancient admixture scenario, although cannot completely exclude it. Overall, our analyses support the hypothesis that C3* Y chromosomes were present in the “First American” ancestral population, and have been lost by drift from most modern populations except the Ecuadorians.

## Figures and Tables

**Supplementary Fig. 1:**
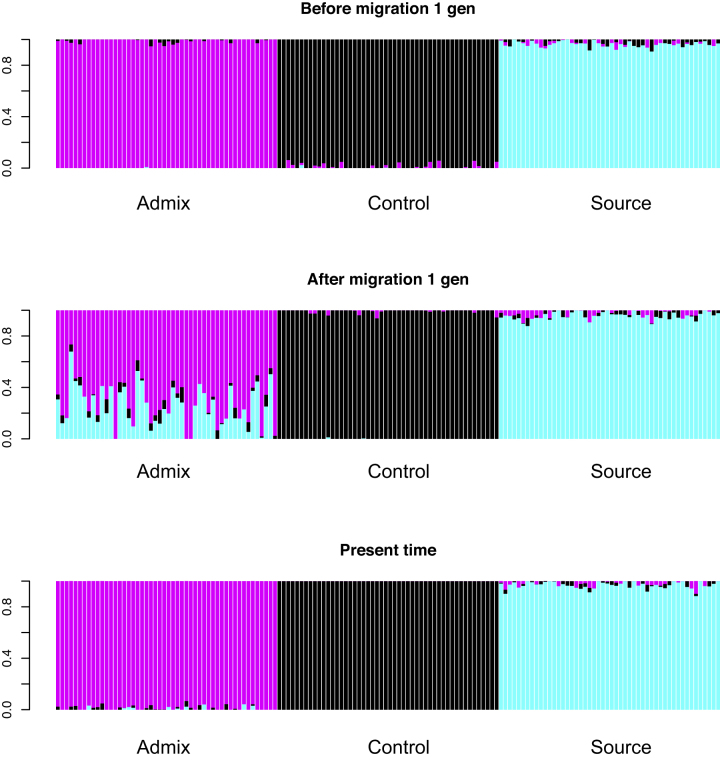
ADMIXTURE analysis of one simulation with migration probability 10% from Source population. The panels show the results before the migration event, immediately after the migration event, and at the present time 6 Ky later.

**Table 1:** Summary of results from the three-population and ALDER tests for migration into the Ecuadorian population.

Test	Number of tests performed	*Z*-score (min–max)	Number of significant tests
Three-population	741	32.25–133.10	0
ALDER	153	0–3.79	0
