# Supplementary material for: Insights into the origin of rare haplogroup C3* Y chromosomes in South America from high-density autosomal SNP genotyping
Source: Forensic Sci Int Genet. 2015 Mar;15:115–20. doi: 10.1016/j.fsigen.2014.11.005 (PMC4312352; doi:10.1016/j.fsigen.2014.11.005)
Supplement: Supplementary file 1 [file mmc4.docx]

**Tables**

**Supplementary Table 1: The Ecuador samples that passed QC and were used for the final analyses**

| Sample_Ids | Region | Population | Sex | Y-Haplogroup |
| --- | --- | --- | --- | --- |
| Ba32 | Ecuador | Kichwa | MALE | Q-M3 |
| Ba47 | Ecuador | Kichwa | FEMALE |  |
| To07 | Ecuador | Kichwa | MALE | Q-M3 |
| To82 | Ecuador | Kichwa | FEMALE |  |
| To83 | Ecuador | Kichwa | MALE | Q-M3 |
| Ba05 | Ecuador | Waorani | MALE | Q-M3 |
| Ba19 | Ecuador | Waorani | FEMALE |  |
| Ba25 | Ecuador | Waorani | MALE | Q-M3 |
| Ba28 | Ecuador | Waorani | FEMALE |  |
| Ba55 | Ecuador | Waorani | MALE | Q-M3 |
| Ba30 | Ecuador | Waorani | MALE | Q-M3 |
| To20 | Ecuador | Waorani | FEMALE |  |
| To31 | Ecuador | Waorani | MALE | Q-M3 |
| To66 | Ecuador | Waorani | MALE | Q-M3 |
| To91 | Ecuador | Waorani | FEMALE |  |
| To64 | Ecuador | Waorani | FEMALE |  |

**Supplementary Table 2: Numbers of samples from each population used for the analyses**

| Population | Sample Size |
| --- | --- |
| Kichwa | 5 |
| Waorani | 11 |
| Adygei | 17 |
| Balochi | 24 |
| Bantu | 19 |
| Basque | 24 |
| Bedouin | 46 |
| BiakaPygmies | 21 |
| Brahui | 25 |
| Burusho | 25 |
| Cambodian | 10 |
| Colombian | 7 |
| Druze | 42 |
| Ecuador | 16 |
| French | 28 |
| Han | 44 |
| Hazara | 22 |
| Japan | 39 |
| Kalash | 23 |
| Karitiana | 14 |
| Makrani | 25 |
| Mandenka | 22 |
| NorthItaly | 20 |
| NorthWestChina | 29 |
| Orcadian | 15 |
| Palestinian | 46 |
| Papuan | 17 |
| Pathan | 22 |
| Pima | 14 |
| Russian | 25 |
| San | 5 |
| Sardinian | 28 |
| Sindhi | 24 |
| SouthChina | 66 |
| Surui | 8 |
| Yakut | 25 |
| Yoruba | 21 |

**Supplementary Table 3. NeON parameter estimation**

| Population | Initial Ne | Final Ne | Growth Model |
| --- | --- | --- | --- |
| Ancestral population (Nanc1) | 7000 | 7000 | Constant |
| Ancestral population (Nanc) | 3000 | 2500 | Exponential decrease |
| Japan (Source) | 4000 | 9500 | Exponential increase |
| Ecuador (Admix and Control) | 1750 | 1400 | Exponential decrease |
